# Achilles Tendon Rupture

**DOI:** 10.5811/westjem.2014.10.24127

**Published:** 2014-11-26

**Authors:** Sean P. Stickles, Larry Friedman, Michael Demarest, Christopher Raio

**Affiliations:** * University of Missouri, Department of Emergency Medicine, Columbia, Missouri*; † Lenox Hill Hospital, Department of Emergency Medicine, New York, New York; ‡ North Shore University Hospital, Department of Emergency Medicine, Manhasset, New York

A 60-year-old man presented to the emergency department complaining of acute onset posterior ankle pain. He reported playing tennis earlier in the afternoon when he suddenly stopped and pivoted, noting a “pop” sensation and pain to the right posterior ankle. The pain was sharp and increased with movement. The patient also experienced difficulty weight bearing and ambulating. There was a palpable defect at the distal end of the expected course of the Achilles tendon and lack of plantar flexion with squeezing the affected calf (Figure A). Point-of-care ultrasound (POCUS) was performed for further evaluation, and noted a discontinuity of the Achilles tendon with retracted proximal and distal ends, consistent with rupture (Figure B; Video). Orthopedics was consulted, and the patient was admitted for operative repair.

Achilles tendon rupture typically occurs by pushing off the weight-bearing foot with knee extended, sudden dorsiflexion of the ankle, or forceful dorsiflexion of a plantar-flexed foot.^1^,^2^ Physical examination may reveal a palpable defect at the tendon injury site, loss of strength with voluntary plantar flexion, increased passive dorsiflexion, and loss of plantar flexion with squeezing of the calf when the patient is lying prone (Simmonds-Thompson test).^3^–^5^ POCUS using a high-frequency transducer has been shown to be effective at visualizing Achilles tendon rupture, noting loss of the tightly arranged fibrillar pattern of the tendon fibers and an area of hypoechogenicity at the site of tendon defect.^6^,^7^ Additional ultrasound findings of tendon rupture include hematoma formation at the site of rupture and posterior acoustic shadowing at the retracted rupture margins.^8^ To avoid misdiagnosis of tendon injury due to the effect of anisotropy as the tendon fibers insert on the calcaneus, one should completely scan through the tendon course and insertion point in different planes.^7^

## Figures and Tables

**Figure A and B:**
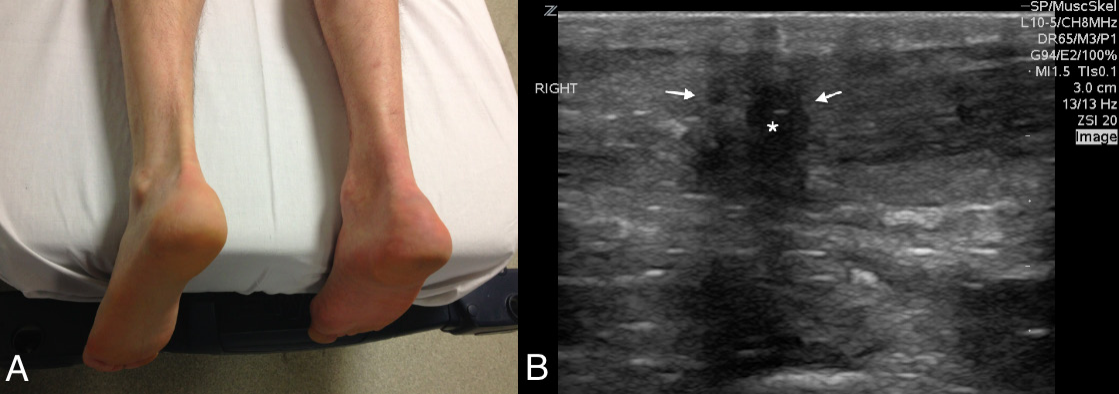
(A) Comparison of both posterior ankles in patient with right posterior Achilles tendon rupture. (B) Longitudinal ultrasound image of the area of pain to the posterior right ankle, noting retracted ends of the Achilles tendon (arrows) and hematoma in-between (*).

**Video:** Ultrasound video of the Achilles tendon using a linear transducer demonstrating retracted proximal and distal tendon ends, separated by a mixed echogenic focus (hematoma), consistent with complete tendon rupture.
